# Quality of life with nivolumab and AVD first‐line treatment of Hodgkin lymphoma in the Phase II GHSG NIVAHL trial

**DOI:** 10.1002/hem3.70485

**Published:** 2026-09-29

**Authors:** Christian Peter Jaworek, Ina Bühnen, Peter Herhaus, Julia Meissner, Karolin Trautmann‐Grill, Teresa Halbsguth, Andrea Kerkhoff, Valdete Schaub, Michael Fuchs, Bastian von Tresckow, Peter Borchmann, Karolin Behringer, Paul Jan Bröckelmann

**Affiliations:** ^1^ Faculty of Medicine and University Hospital of Cologne, Department I of Internal Medicine, Center for Integrated Oncology Aachen Bonn Cologne Duesseldorf (CIO ABCD) University of Cologne Cologne Germany; ^2^ German Hodgkin Study Group (GHSG) Cologne Germany; ^3^ Department of Internal Medicine III TUM University Hospital, Technical University of Munich, School of Medicine and Health Munich Germany; ^4^ Medicine V University of Heidelberg Heidelberg Germany; ^5^ University Hospital Carl Gustav Carus Technische Universität Dresden Dresden Germany; ^6^ Department of Oncology Universitätsklinikum Frankfurt, Johann Wolfgang Goethe‑Universität Frankfurt am Main Frankfurt am Main Germany; ^7^ Medical Clinic A, Universitätsklinikum Münster, Westfälische Wilhelms‑Universität Münster Münster Germany; ^8^ Internal Medicine VIII Universitätsklinikum Tübingen, Eberhard Karls Universität Tübingen Tübingen Germany; ^9^ Department of Hematology and Stem Cell Transplantation, West German Cancer Center, University Hospital Essen University of Duisburg‐Essen Essen Germany

First‐line treatment of classic Hodgkin lymphoma (HL) is undergoing a shift towards early incorporation of targeted agents. The anti‐PD1‐based regimen nivolumab plus doxorubicin, vinblastine and dacarbazine (N‐AVD) is now approved for patients with advanced‐stage disease and also considered a treatment option for intermediate‐stage HL.^1^, ^2^, ^3^ Because cure rates are high and many HL patients are young, longitudinal quality of life (QoL), fatigue, and return to work are central to shared decision‐making. To this end, the QoL impact of anti‐PD1‐based first‐line treatment remains poorly described.^4^, ^5^

To address this knowledge gap, we herein report the preplanned longitudinal QoL analysis of the randomized Phase II GHSG NIVAHL trial (NCT03004833). In NIVAHL, 109 patients aged 18–60 years with intermediate‐stage HL received cumulatively four cycles of N‐AVD (concomitant, Group A; or sequential, Group B), each followed by 30 Gy involved‐site radiotherapy (IS‐RT).^1^, ^2^ Patients providing separate informed consent completed the validated EORTC QLQ‐C30 and QLQ‐FA12 questionnaires at enrollment, after systemic treatment, after IS‐RT, and at 1‐ and 3‐year follow‐up (FU).^6^, ^7^ Functional, symptom, and global health status (GHS) scores were age‐ and sex‐standardized to German general population reference values, with zero indicating no difference from the reference population.^8^ As previously established, fatigue scores of 30 or higher were considered clinically relevant.^9^ Differences from the reference population and changes over time were considered statistically significant if the corresponding 95% CI excluded zero. Time to recovery from fatigue (TTR‐F; interval from end of treatment [EOT] to the first assessment with a fatigue score below 30) and time to return to work (TTR‐W; interval from EOT to the first documented return to work or education among patients reporting employment or education before diagnosis) were analyzed using time‐to‐event methods, with multivariable Cox models adjusted for age and sex. In an exploratory analysis, the impact of immune‐related hypothyroidism (irHypothyroidism), the most frequent chronic immune‐related adverse event (irAE), on QoL domains was assessed. Analyses were performed in SAS 9.4 (SAS Institute, Cary, NC, USA).

Of 109 patients, 98 (90%) were evaluable for the present analysis, contributing 397 questionnaires (median 4 per patient) with high completion (QLQ‐C30 93%, QLQ‐FA12 97%) and declining availability over time (Figures S1 and S2). The median age was 27 years (range 18–60), 60% were female, 74% had an ECOG of 0, and 77% presented with Stage IIA disease. As expected in this trial for patients with intermediate‐stage HL, at least one of the GHSG risk factors was present in all patients, with ≥3 nodal areas most common (Table S1). Baseline life situation, including educational level and employment, is detailed in Table S2. Patients with and without an evaluable late assessment were largely comparable in age, sex, stage, educational level and treatment arm and had virtually identical FU, although those contributing 3‐year data reported lower baseline fatigue (Table S3).

Compared to the age‐ and sex‐matched German general population,^8^ HL patients in NIVAHL reported impaired GHS already at baseline relative to reference values (mean difference −26, 95% CI −30 to −21) and worsened further on treatment (−35, 95% CI −40 to −30; Figure 1). Baseline symptom burden was highest for fatigue (+22), appetite loss (+17), financial impact (+15), sleep disturbance (+13), and dyspnea (+10), whereas functioning impairment was most pronounced for emotional (−23) and role functioning (−15). On treatment, fatigue (+39) and most symptom and functioning scales deteriorated, including nausea and vomiting despite mandatory dexamethasone‐containing prophylaxis (+22), with role functioning showing the steepest on‐treatment decline (−41).

**Figure 1:**
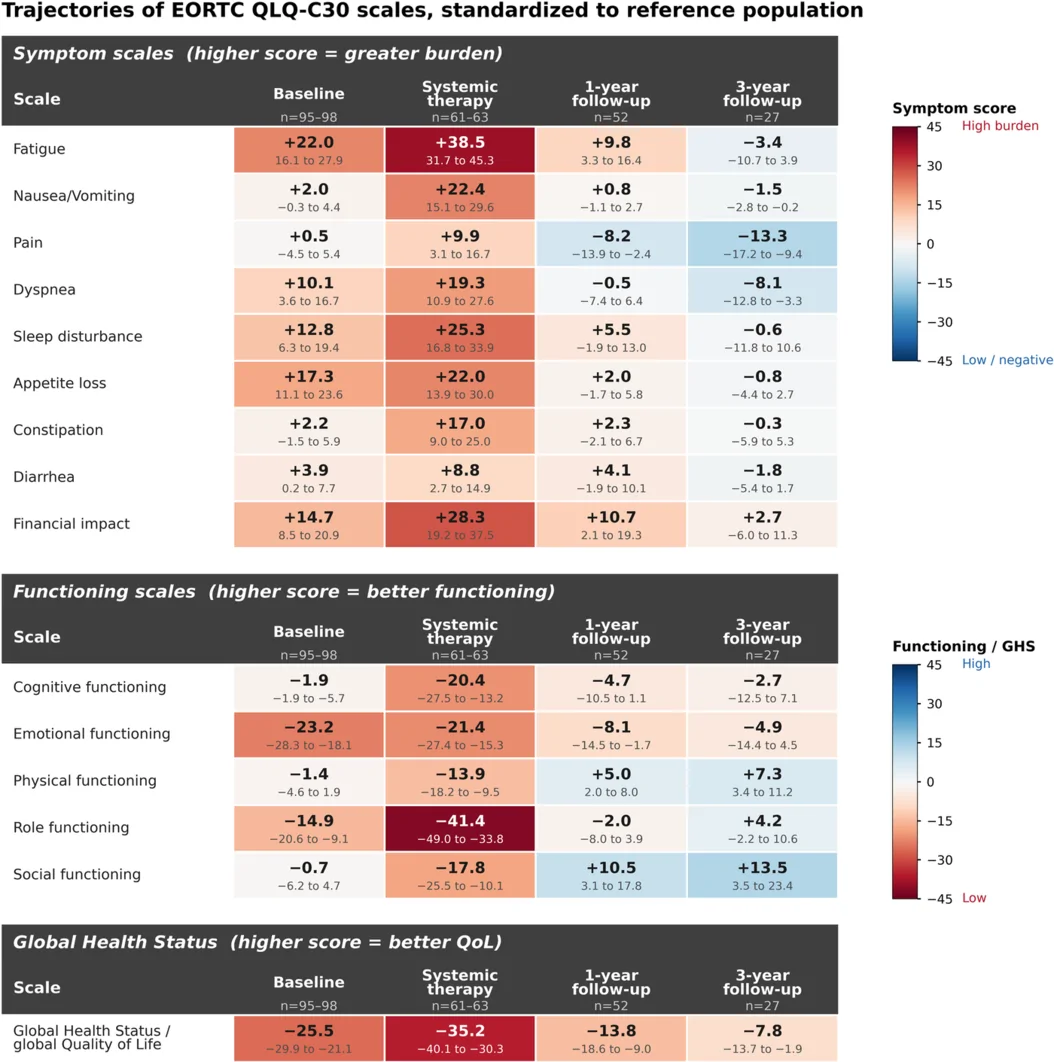
**Trajectories of EORTC QLQ‐C30 scales, standardized to reference population.** Each cell shows mean score (bold) and 95% confidence intervals (bottom). Symptom scales: blue = low/negative burden, red = high burden. Functioning and global health status (GHS): red = poor/low, blue = good/high. Sample sizes at baseline (95–98) and after systemic therapy (61–63) are given as range since the number of patients with fully evaluable questionnaires differed per scale (Tables S4–S6). QoL, quality of life.

After EOT, however, most domains improved substantially compared to baseline and on‐treatment status. Importantly, most domains such as for example GHS (−14 at 1 year; −8 at 3 years) or fatigue (+10 at 1 year; −3 at 3 years; Figures 1, S3 and S4; unadjusted scores in Tables S4–S6) approximated reference values during FU. In Cox regression, baseline scores were consistently associated with the corresponding 1‐ and 3‐year scores, whereas effects of age and sex were small and domain‐specific (Tables S7–S10).

A more detailed analysis of fatigue was conducted from the QLQ‐FA12 measurements. Physical fatigue showed the highest burden and the most pronounced on‐treatment deterioration before improvement occurred during FU. Cognitive fatigue increased transiently and returned to baseline, while emotional fatigue improved steadily (Figure S5, Table S11). In an exploratory comparison, GHS and fatigue trajectories did not differ markedly between the concomitant and sequential treatment groups (Figure S6, Table S12). Median TTR‐F (*n* = 79) was 12.8 months (95% CI 7.3–20.3) and median TTR‐W (*n* = 54, among patients employed or in education before diagnosis) was 3.8 months (1.4–8.0), with most patients recovering from severe fatigue and being able to return to work or education (Figure 2). In Cox regression analyses, higher baseline fatigue was associated with delayed fatigue recovery, and TTR‐W tended to be shorter in men, whereas neither endpoint differed between treatment arms (Table S13).

**Figure 2:**
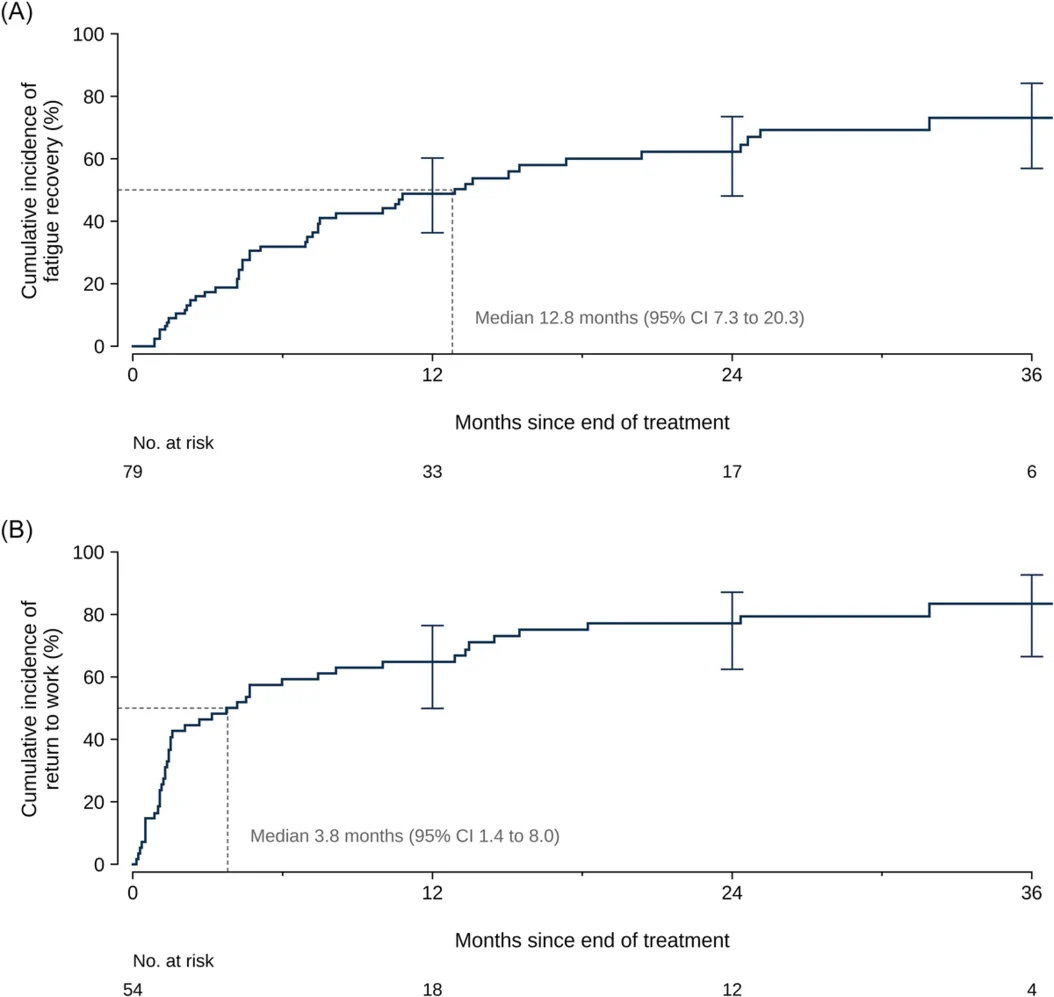
**Time to recover from fatigue (TTR‐F, A) and time to return to work (TTR‐W, B).** CI, confidence interval; EOT, end of treatment.

Treatment‐related irHypothyroidism developed in 21 of 98 patients (21%). On‐treatment impairment of QoL and symptoms, particularly fatigue, sleep disturbance and appetite loss, appeared more pronounced in affected patients compared to those without irHypothyroidism. Overall, no consistent inter‐group differences were apparent at 1 and 3 years, and the low numbers at 3 year FU (*n* = 4 with irHypothyroidism vs. 23 without) preclude firm conclusions. This comparison was exploratory and hypothesis‐generating (Figure S7, Tables S14 and S15).

To our knowledge, this is the first comprehensive longitudinal characterization of patient‐reported QoL domains, including fatigue, other symptoms, functioning and return to work with anti‐PD1‐based first‐line HL treatment. The analysis benefits from high patient participation, relatively high longitudinal completeness and standardization to national age‐ and sex‐matched reference values, extending prior GHSG longitudinal QoL data from chemotherapy‐based regimens^10^, ^11^ to the anti‐PD1 setting. The on‐treatment increase of fatigue parallels a preliminary signal of higher fatigue with N‐AVD than with brentuximab vedotin plus AVD in the Phase III SWOG S1826 trial,^12^, ^13^ suggesting a possible class effect of anti‐PD1 antibodies in the HL first‐line population. Of note, S1826 differs from NIVAHL in enrolling adolescents and adults aged 12 and older with advanced‐stage (III–IV) disease treated with six cycles and virtually no consolidative radiotherapy, whereas NIVAHL enrolled patients aged 18–60 years with intermediate‐stage disease. This observation warrants further exploration and ideally confirmation in larger series, since it may justify early fatigue‐directed intervention such as exercise, cognitive behavioral therapy and psychosocial support.^14^ Notably, disease control in NIVAHL was near‐universal (92% complete remission, 3‐year progression‐free survival 99% and overall survival 100%,^1^, ^2^ so the observed QoL trajectories reflect outcomes under effective first‐line therapy rather than being confounded by treatment failure. Reassuringly, QoL improved during FU with reference values approximated for most domains, consistent with patterns observed after other highly effective contemporary regimens.^15^, ^16^, ^17^ These data enable more granular patient counseling and support survivorship‐focused care rather than the assumption of permanent impairment. Because deterioration peaks on or at the EOT, this window appears key for proactive symptom monitoring and symptom‐oriented interventions. Limitations of the present analysis include the absence of a non‐anti‐PD1 control arm, possible under‐reporting of less frequent irAE, and attrition at later FU.^18^ As patients contributing 3‐year data reported lower baseline fatigue, missingness cannot be assumed to be at random and late estimates may be somewhat optimistic.^19^ The ongoing GHSG INDIE trial (NCT04837859), with collection of longitudinal PRO data including PRO‐CTCAE and a more detailed irAE assessment as well as the pending more detailed QoL analysis from the S1826 trial will hopefully address some of the remaining uncertainties in the future.

Collectively, the data and findings reporting herein help counsel patients on the expected QoL trajectories with transient worsening of QoL incl. fatigue, before meaningful recovery beyond baseline status during FU.

## AUTHOR CONTRIBUTIONS

**Christian Peter Jaworek:** Writing—original draft; writing—review and editing; formal analysis. **Ina Bühnen:** Writing—review and editing; formal analysis; **Peter Herhaus:** Writing—review and editing; investigation; data curation. **Julia Meissner:** Data curation; investigation; writing—review and editing. **Karolin Trautmann‐Grill:** Data curation; investigation; writing—review and editing. **Teresa Halbsguth:** Investigation; writing—review and editing; data curation. **Andrea Kerkhoff:** Data curation; investigation; writing—review and editing. **Valdete Schaub:** Data curation; investigation; writing—review and editing. **Michael Fuchs:** Validation; writing—review and editing. **Bastian von Tresckow:** Writing—review and editing; investigation; data curation. **Peter Borchmann:** Conceptualization; writing—review and editing; investigation; data curation. **Karolin Behringer:** Conceptualization; writing—review and editing. **Paul Jan Bröckelmann:** Writing—review and editing; conceptualization; methodology; project administration; supervision; investigation; data curation; writing—original draft.

## CONFLICT OF INTEREST STATEMENT

P.J.B. is an advisor or consultant for Hexal, Merck Sharp & Dohme (MSD), Need Inc., Stemline, Takeda, and Therakos; holds stock options in Need AI, has received honoraria from AstraZeneca, BeOne, Bristol‐Myers Squibb/Celgene (BMS), Lilly, MSD, Need AI, Roche, Stemline, and Takeda; and reports research funding from BeiGene (institution), BMS (institution), MSD (institution), and Takeda (institution). A.K. has received honoraria and travel support from AbbVie, Alexion, Amgen, AstraZeneca, BeiGene, BMS, Lilly, Novartis, Roche, Recordati, Sobi, and Takeda. B.v.T. is an advisor or consultant for AbbVie, Allogene, Amgen, BMS/Celgene, Cerus, Gilead Kite, Incyte, IQVIA, Janssen‐Cilag, Lilly, MSD, Miltenyi, Novartis, Noscendo, Pentixapharm, Pfizer, Pierre Fabre, Qualworld, Regeneron, Roche, Serb, Sobi, and Takeda; has received honoraria from AbbVie, AstraZeneca, BMS/Celgene, Gilead Kite, Incyte, Janssen‐Cilag, Lilly, MSD, Novartis, Roche, Serb, and Takeda; reports research funding from Esteve (institution), MSD (institution), Novartis (institution), and Takeda (institution); reports travel support from AbbVie, AstraZeneca, Gilead Kite, Janssen‐Cilag, Lilly, MSD, Pierre Fabre, Roche, Takeda, and Novartis; and is member of steering committees for Regeneron (institution) and Takeda. All other authors do not declare any potential conflicts of interest.

## ETHICS STATEMENT

The NIVAHL trial was approved by the Ethics Committee of the Faculty of Medicine of the University of Cologne, Cologne, Germany (reference number 16‐444), and by the competent German federal authority (Paul‐Ehrlich‐Institute). It was conducted in accordance with the Declaration of Helsinki and the ICH Good Clinical Practice guidelines. All patients provided written informed consent before enrollment, including consent to the collection and analysis of patient‐reported outcome data. The trial is registered at ClinicalTrials.gov (NCT03004833) and EudraCT (2016‐002626‐37).

## FUNDING

The NIVAHL trial was sponsored by the University of Cologne and was supported by funding and supply of nivolumab from Bristol Myers Squibb. The funder had no role in the design of the trial, the present analysis, or in the interpretation of the data. Open Access funding enabled and organized by Projekt DEAL.

## Supporting information

Supporting Information.

Supporting Information.

## Data Availability

The data that support the findings of this study are available on request from the corresponding author. The data are not publicly available due to privacy or ethical restrictions.

## References

[1] Bröckelmann PJ , Goergen H , Keller U , et al. Efficacy of nivolumab and AVD in early‐stage unfavorable classic Hodgkin lymphoma: the randomized phase 2 German Hodgkin Study Group NIVAHL trial. JAMA Oncol. 2020;6(6):872‐880. 10.1001/jamaoncol.2020.0750 PMC7193521 32352505

[2] Bröckelmann PJ , Bühnen I , Meissner J , et al. Nivolumab and doxorubicin, vinblastine, and dacarbazine in early‐stage unfavorable Hodgkin lymphoma: final analysis of the randomized German Hodgkin Study Group Phase II NIVAHL trial. J Clin Oncol. 2023;41(6):1193‐1199. 10.1200/JCO.22.02355 36508302

[3] Jaworek CP , Bröckelmann PJ . Feasibility and safety of anti‐PD1 first‐line treatment of classic Hodgkin lymphoma: do we see the full picture? Blood. 2025;146(26):3145‐3152. 10.1182/blood.2025029935 41175363

[4] Bröckelmann PJ . Hodgkin lymphoma: great progress with room for improvement. Nat Rev Clin Oncol. 2025;22(6):379‐381. 10.1038/s41571-025-01012-2 40065096

[5] Bröckelmann PJ , Scheffer Cliff ER , Iacoboni G , et al. Beyond maximum grade: tolerability of immunotherapies, cellular therapies, and targeted agents in haematological malignancies. Lancet Haematol. 2025;12(6):e470‐e481. 10.1016/S2352-3026(25)00051-1 40447355

[6] Aaronson NK , Ahmedzai S , Bergman B , et al. The European Organization for Research and Treatment of Cancer QLQ‐C30: a quality‐of‐life instrument for use in international clinical trials in oncology. J Natl Cancer Inst. 1993;85(5):365‐376. 10.1093/jnci/85.5.365 8433390

[7] Weis J , Tomaszewski KA , Hammerlid E , et al. International psychometric validation of an EORTC quality of life module measuring cancer related fatigue (EORTC QLQ‐FA12). J Natl Cancer Inst. 2017;109(5):djw273. 10.1093/jnci/djw273 28376231

[8] Hinz A , Singer S , Brähler E . European reference values for the quality of life questionnaire EORTC QLQ‐C30: results of a German investigation and a summarizing analysis of six European general population normative studies. Acta Oncol. 2014;53(7):958‐965. 10.3109/0284186X.2013.879998 24456505

[9] Ferdinandus J , Müller H , Jablonski J , et al. BrECADD vs BEACOPP and health‐related quality of life in advanced‐stage Hodgkin lymphoma. JAMA Oncol. 2026;12(4):415‐417. 10.1001/jamaoncol.2025.6327 PMC12878636 41642616

[10] Kreissl S , Müller H , Goergen H , et al. Health‐related quality of life in patients with Hodgkin lymphoma: a longitudinal analysis of the German Hodgkin Study Group. J Clin Oncol. 2020;38(25):2839‐2848. 10.1200/JCO.19.03160 32574114

[11] Behringer K , Müller H , Görgen H , et al. Sexual quality of life in Hodgkin lymphoma: a longitudinal analysis by the German Hodgkin Study Group. Br J Cancer. 2013;108(1):49‐57. 10.1038/bjc.2012.550 PMC3553532 23321510

[12] Herrera AF , LeBlanc M , Castellino SM , et al. Nivolumab + AVD in advanced‐stage classic Hodgkin's lymphoma. N Engl J Med. 2024;391(15):1379‐1389. 10.1056/NEJMoa2405888 PMC11488644 39413375

[13] Davison K , Unger J , Li H , et al. The influence of age on patient‐reported outcomes (PROs) endpoints on the S1826 cross‐network Phase III trial of advanced‐stage, classic Hodgkin lymphoma (cHL). Blood. 2025;146(suppl 1):2738. 10.1182/blood-2025-2738

[14] Esser P , Müller H , Borchmann P , et al. Web‐based cognitive‐behavioral therapy to reduce severe cancer‐related fatigue among survivors of Hodgkin lymphoma: a feasibility study. J Clin Psychol Med Settings. 2023;30(4):856‐865. 10.1007/s10880-023-09944-6 PMC9943038 36808045

[15] Borchmann P , Ferdinandus J , Schneider G , et al. Assessing the efficacy and tolerability of PET‐guided BrECADD versus eBEACOPP in advanced‐stage, classical Hodgkin lymphoma (HD21): a randomised, multicentre, parallel, open‐label, phase 3 trial. Lancet. 2024;404(10450):341‐352. 10.1016/S0140-6736(24)01315-1 38971175

[16] Bröckelmann PJ , Müller H , Fuchs M , et al. Correlation between progression‐free and overall survival in patients with Hodgkin lymphoma: a comprehensive analysis of individual patient data from randomized German Hodgkin Study Group (GHSG) trials. Ann Oncol. 2025;36(4):393‐402. 10.1016/j.annonc.2024.12.009 39706337

[17] Ferdinandus J , Kaul H , Fosså A , et al. Positron emission tomography‐guided brentuximab vedotin, etoposide, cyclophosphamide, doxorubicin, dacarbazine, and dexamethasone in older patients with advanced‐stage classic Hodgkin lymphoma: a prospective, multicenter, single‐arm, phase II cohort of the German Hodgkin Study Group HD21 trial. J Clin Oncol. 2025;43(27):2974‐2985. 10.1200/JCO-25-00439 40674676

[18] Bröckelmann PJ . Considerations for anti‐PD1‐based first‐line treatment of Hodgkin lymphoma. Br J Haematol. 2024;205(1):22‐24. 10.1111/bjh.19534 38757338

[19] Billingham L , Abrams K , Jones D . Methods for the analysis of quality‐of‐life and survival data in health technology assessment. Health Technol Assess (Rockv). 1999;3(10):1‐152. 10.3310/hta3100 10627631

