# Supplementary material for: Quality of life with nivolumab and AVD first‐line treatment of Hodgkin lymphoma in the Phase II GHSG NIVAHL trial
Source: Hemasphere. 2026 Sep 29;10(10):e70485. doi: 10.1002/hem3.70485 (PMC13622444; doi:10.1002/hem3.70485)
Supplement: Supplementary file 1 [file HEM3-10-e70485-s002.docx]

**Supplementary Material**

**Supplementary Figures**

***Supplementary Figure S1:* *Consort charts for the analysis populations***

*(A) Quality of life (QoL) analysis population; (B) Time to recover from fatigue (TTR-F) analysis population; (C) Time to return to work (TTR-W) analysis population. Abbreviations: QoL: quality of life, EOT: end of treatment.*

***Supplementary Figure S2: Availability of QoL questionnaires over time***

*Number of patients (N=98) with an evaluable questionnaire at each time point. Abbreviations: IS-RT: involved-site radiotherapy; FU: follow-up.*

***Supplementary Figure S3: QoL Trajectories, standardized to reference population***

**A**

**B**

*(A): Global health status (GHS), a value of zero indicates no difference between study population and reference population. (B) Fatigue, a value of zero indicates no difference between study population and reference population. Positive values indicate higher symptom burden. Mean values, 95% confidence interval. Abbreviations: Nivo-AVD: End of systemic treatment with 4 cycles of nivolumab with doxorubicin, vinblastine and dacarbazine; RT: radiotherapy, FU: follow-up.*

***Supplementary Figure S4: Unadjusted QoL trajectories***

**A**

**B**

*(A): Global health status (GHS), a high value indicates good QoL. (B) Fatigue, a high value indicates fatigue with the dashed line representing the cut-off for severe fatigue (30). Mean values, 95% confidence interval. Abbreviations: Nivo-AVD: End of systemic treatment with 4 cycles of nivolumab with doxorubicin, vinblastine and dacarbazine; RT: radiotherapy, FU: follow-up.*

***Supplementary Figure S5: Trajectories of physical, emotional and cognitive fatigue by FA12***

*Abbreviations: Nivo-AVD: End of systemic treatment with 4 cycles of nivolumab with doxorubicin, vinblastine and dacarbazine; RT: radiotherapy, FU: follow-up.*

***Supplementary Figure S6: Unadjusted QoL trajectories by treatment arm***

**A**

**B**

*(A): Global health status (GHS), a high value indicates good QoL. (B) Fatigue, a high value indicates high fatigue. Mean values, 95% confidence interval. Abbreviations: Nivo-AVD: nivolumab with doxorubicin, vinblastine and dacarbazine; RT: radiotherapy; FU: follow-up.*

***Supplementary Figure S7: QoL Trajectories in patients with and without immune-related Hypothyroidism, normalized to the reference population***

**A**

**B**

*(A): Global health status (GHS) in patients with (yes) and without (no) irHypothyroidism, a value of zero indicates no difference between study population and reference population. (B) Fatigue in patients with (yes) and without (no) irHypothyroidism, a value of zero indicates no difference between study population and reference population. Positive values indicate higher symptom burden. Mean values, 95% confidence interval. Abbreviations: Nivo-AVD: nivolumab with doxorubicin, vinblastine and dacarbazine; RT: radiotherapy, FU: follow-up.*

**Supplementary Material**

**Supplementary Tables**

***Supplementary Table S1: Patient characteristics of the HR-QoL NIVAHL cohort***

|  | **Concomitant**  N=50 | **Sequential**  N=48 | **Total**  N=98 |
| --- | --- | --- | --- |
| **Age**  Median (range) | 26 (18-57) | 29 (18-60) | 27 (18-60) |
| **Sex**  Female  Male | 31 (62%)  19 (38%) | 28 (58%)  20 (42%) | 59 (60%)  39 (40%) |
| **Performance status**  ECOG=0  ECOG=1 | 37 (74%)  13 (26%) | 36 (75%)  12 (25%) | 73 (74%)  25 (26%) |
| **Ann Arbor stage**  IA  IB  IIA  IIB | 2 (4%)  0 (0%)  38 (76%)  10 (20%) | 1 (2%)  1 (2%)  37 (77%)  9 (19%) | 3 (3%)  1 (1%)  75 (77%)  19 (20%) |
| **GHSG risk factors**  Large mediastinal mass  Extranodal involvement  Elevated ESR  ≥3 nodal areas | 7 (14%)  7 (14%)  21 (42%)  37 (74%) | 12 (25%)  4 (8%)  23 (48%)  33 (69%) | 19 (19%)  11 (11%)  44 (45%)  70 (71%) |
| **Other risk factors**  Infradiaphragmal involvement  Bulky disease | 2 (4%)  28 (56%) | 0 (0%)  36/47 (77%) | 2 (2%)  64/97 (66%) |

*Abbreviations: HR-QoL: Health-related quality of life, ECOG: Eastern cooperative group performance status, ESR: erythrocyte sedimentation rate.*

***Supplementary Table S2: Life situation prior treatment, of available patients***

|  | **Concomitant**  N=38 | **Sequential**  N=34 | **Total**  N=72 |
| --- | --- | --- | --- |
| **Siblings**  Yes  No | 33 (87%)  5 (13%) | 28 (82%)  6 (18%) | 61 (85%)  11 (15%) |
| **Number of siblings**  Median (range) | 2 (1-5) | 1 (1-7) | 1 (1-7) |
| **Marital status**  Unmarried  Married  Divorced / separated | 26 (68%)  11 (29%)  1 (3%) | 22 (65%)  10 (29%)  2 (6%) | 48 (67%)  21 (29%)  3 (4%) |
| **Relationship status**  Single  Temporary relationship  Steady relationship  Other | 13 (34%)  2 (5%)  22 (58%)  1 (1%) | 8 (24%)  0 (0%)  23 (68%)  3 (9%) | 21 (29%)  2 (3%)  45 (63%)  4 (6%) |
| **Highest level of education**  No secondary school diploma  Lower secondary school diploma  Upper secondary school diploma  University-level education | 1 (3%)  16 (42%)  9 (24%)  12 (32%) | 1 (3%)  11 (32%)  11 (32%)  11 (32%) | 2 (3%)  27 (38%)  20 (28%)  23 (32%) |
| **Employment status**  Full-time employment  Part-time employment  Education / apprenticeship  Unable to work or retired | 23 (61%)  4 (11%)  8 (21%)  3 (8%) | 21 (62%)  5 (15%)  8 (24%)  0 (0%) | 44 (61%)  9 (13%)  16 (22%)  3 (4%) |

***Supplementary Table S3: Characteristics of patients with and without an evaluable follow-up assessment***

|  | **Year 1 completer**  N=52 | **Year 1 non-completer**  N=46 | **Year 3 completer**  N=27 | **Year 3 non-completer**  N=71 |
| --- | --- | --- | --- | --- |
| **Age**  Median (range) | 27 (18-60) | 27 (18-57) | 28 (18-60) | 27 (18-57) |
| **Sex**  Female  Male | 32 (62%)  20 (38%) | 27 (59%)  19 (41%) | 15 (56%)  12 (44%) | 44 (62%)  27 (38%) |
| **Performance status**  ECOG=0  ECOG=1 | 39 (75%)  13 (25%) | 34 (74%)  12 (26%) | 25 (93%)  2 (7%) | 48 (68%)  23 (32%) |
| **Ann Arbor stage**  IA  IB  IIA  IIB | 1 (2%)  1 (2%)  43 (83%)  7 (13%) | 2 (4%)  0 (0%)  32 (70%)  12 (26%) | 1 (4%)  1 (4%)  22 (81%)  3 (11%) | 2 (3%)  0 (0%)  53 (75%)  16 (23%) |
| **GHSG risk factors**  Large mediastinal mass  Extranodal involvement  Elevated ESR  ≥3 nodal areas | 11 (21%)  1 (2%)  22 (42%)  35 (67%) | 8 (17%)  10 (22%)  22 (48%)  35 (76%) | 5 (19%)  1 (4%)  11 (41%)  19 (70%) | 14 (20%)  10 (14%)  33 (46%)  51 (72%) |
| **Other risk factors**  Infradiaphragmal involvement  Bulky disease | 2 (4%)  35/51 (69%) | 0 (0%)  29 (63%) | 1 (4%)  18/26 (69%) | 0 (0%)  46 (65%) |
| **Treatment arm**  Concomitant (Arm A)  Sequential (Arm B) | 28 (54%)  24 (46%) | 22 (48%)  24 (52%) | 12 (44%)  15 (56%) | 38 (54%)  33 (46%) |
| **Median follow up**  Months | 40.3 | 40.2 | 41.0 | 40.1 |
| **Highest level of education**  No secondary school diploma  Lower secondary school diploma  Upper secondary school diploma  University-level education | 1/38 (3%)  14/38 (37%)  8/38 (21%)  15/38 (39%) | 1/34 (3%)  13/34 (38%)  12/34 (35%)  8/34 (24%) | 1/22 (5%)  7/22 (32%)  8/22 (36%)  6/22 (27%) | 1/50 (2%)  20/50 (40%)  12/50 (24%)  17/50 (34%) |
| **Baseline employment status**  Full-time employment  Part-time employment  Education / apprenticeship  Unable to work or retired | 22/38 (58%)  6/38 (16%)  8/38 (21%)  2/38 (5%) | 22/34 (65%)  3/34 (9%)  8/34 (24%)  1/34 (3%) | 12/22 (55%)  4/22 (18%)  6/22 (27%)  0/22 (0%) | 32/50 (64%)  5/50 (10%)  10/50 (20%)  3/50 (6%) |
| **GHS and fatigue scores**  Baseline GHS, mean (95% CI)  Baseline fatigue, mean (95% CI)  On-treatment fatigue, mean (95% CI) | 67.9 (62.1-73.8)  35.3 (27.1-43.4)  52.5 (44.6-60.4) | 60.1 (53.3-66.8)  44.2 (35.4-53.0)  62.1 (48.8-75.4) | 70.1 (63.0-77.1)  30.0 (20.3-39.8)  48.9 (37.0-60.7) | 62.1 (56.6-67.6)  43.0 (35.7-50.3)  59.3 (50.8-67.8) |

*Patients are classified as completers if an evaluable QoL questionnaire was available at the respective follow-up assessment. Percentages are column percentages. Quality of life scores are means with 95% confidence intervals. Abbreviations: CI: confidence interval; ECOG: Eastern Cooperative Oncology Group performance status; ESR: erythrocyte sedimentation rate; GHS: global health status; GHSG: German Hodgkin Study Group.*

***Supplementary Table S4: EORTC QLQ-C30 global health status, unadjusted***

| **At inclusion** | | | **After N-AVD** | | | **1 year FU** | | | **3 year FU** | | |
| --- | --- | --- | --- | --- | --- | --- | --- | --- | --- | --- | --- |
| **n** | **mean** | **95% CI** | **n** | **mean** | **95% CI** | **n** | **mean** | **95% CI** | **n** | **mean** | **95% CI** |
| 95 | 64.4 | 60.0-68.8 | 62 | 54.7 | 49.8-59.6 | 52 | 76.1 | 71.3-80.9 | 27 | 82.1 | 76.2-88 |

***Supplementary Table S5: EORTC QLQ-C30 functioning scales, unadjusted***

|  | **At inclusion** | | | **After N-AVD** | | | **1 year FU** | | | **3 year FU** | | |
| --- | --- | --- | --- | --- | --- | --- | --- | --- | --- | --- | --- | --- |
|  | **n** | **mean** | **95% CI** | **n** | **mean** | **95% CI** | **n** | **mean** | **95% CI** | **n** | **mean** | **95% CI** |
| Cognitive functioning | 97 | 85.4 | 81.6-89.2 | 62 | 66.9 | 59.8-74.1 | 52 | 82.6 | 76.9-88.3 | 27 | 84.6 | 74.8-94.4 |
| Emotional functioning | 97 | 62.9 | 57.8-68 | 62 | 64.7 | 58.7-70.8 | 52 | 78 | 71.6-84.4 | 27 | 81.2 | 71.7-90.6 |
| Physical functioning | 98 | 87.6 | 84.4-90.9 | 63 | 75.1 | 70.8-79.5 | 52 | 94 | 91-97 | 27 | 96.3 | 92.4-100.2 |
| Role functioning | 98 | 74.1 | 68.4-79.9 | 63 | 47.6 | 40-55.2 | 52 | 87 | 81-92.9 | 27 | 93.2 | 86.8-99.6 |
| Social functioning | 97 | 74.1 | 68.6-79.5 | 62 | 57 | 49.3-64.7 | 52 | 85.3 | 77.9-92.6 | 27 | 88.3 | 78.3-98.2 |

***Supplementary Table S6: EORTC QLQ-C30 symptom scales, unadjusted***

|  | **At inclusion** | | | **After N-AVD** | | | **1 year FU** | | | **3 year FU** | | |
| --- | --- | --- | --- | --- | --- | --- | --- | --- | --- | --- | --- | --- |
|  | **n** | **mean** | **95% CI** | **n** | **mean** | **95% CI** | **n** | **mean** | **95% CI** | **n** | **mean** | **95% CI** |
| Fatigue | 97 | 39.4 | 33.5-45.3 | 62 | 55.9 | 49.1-62.7 | 52 | 27.2 | 20.7-33.8 | 27 | 14 | 6.7-21.3 |
| Nausea and vomiting | 97 | 4.1 | 1.8-6.5 | 62 | 24.5 | 17.2-31.7 | 52 | 2.9 | 1-4.8 | 27 | 0.6 | -0.7-1.9 |
| Pain | 98 | 18.4 | 13.4-23.3 | 63 | 27.8 | 21-34.6 | 52 | 9.7 | 4-15.5 | 27 | 4.6 | 0.7-8.5 |
| Dyspnea | 98 | 23.1 | 16.6-29.7 | 62 | 32.3 | 23.9-40.6 | 52 | 12.5 | 5.6-19.4 | 27 | 4.9 | 0.2-9.7 |
| Insomnia | 98 | 28.2 | 21.7-34.8 | 64 | 40.6 | 32.2-49.1 | 53 | 21.2 | 13.8-28.5 | 27 | 14.8 | 3.6-26 |
| Appetite loss | 97 | 20.6 | 14.4-26.9 | 62 | 25.3 | 17.2-33.3 | 52 | 5.3 | 1.6-9.1 | 27 | 2.5 | -1.1-6 |
| Constipation | 97 | 6.2 | 2.5-9.9 | 62 | 21 | 13-29 | 52 | 6.3 | 1.9-10.7 | 27 | 3.7 | -2-9.3 |
| Diarrhea | 97 | 8.2 | 4.5-12 | 61 | 13.1 | 7-19.2 | 52 | 8.4 | 2.4-14.4 | 27 | 2.5 | -1.1-6 |
| Financial difficulties | 97 | 21.3 | 15.1-27.5 | 62 | 35 | 25.8-44 | 52 | 17.3 | 8.7-25.9 | 27 | 9.3 | 0.6-17.9 |

***Supplementary Table S7: Regression analysis for EORTC QLQ-C30 functioning scales at 1 year FU***

|  | **At inclusion** | | | **Male sex** | | | **Age** | | |
| --- | --- | --- | --- | --- | --- | --- | --- | --- | --- |
|  | **b** | **β** | **95% CI** | **b** | **β** | **95% CI** | **b** | **β** | **95% CI** |
| Cognitive functioning | 0.59 | 0.56 | 0.34-0.83 | 0.07 | 0.00 | -10.28-10.42 | -0.42 | -0.23 | -0.86-0.02 |
| Emotional functioning | 0.34 | 0.37 | 0.1-0.59 | 2.2 | 0.05 | -10.08-14.48 | -0.48 | -0.24 | -1.02-0.05 |
| Physical functioning | 0.09 | 0.12 | -0.1-0.28 | 2.36 | 0.11 | -3.36-8.09 | -0.42 | -0.44 | -0.66 to -0.17 |
| Role functioning | 0.17 | 0.18 | -0.07-0.42 | 6.32 | 0.15 | -5.5-18.14 | -0.57 | -0.31 | -1.07 to -0.06 |
| Social functioning | 0.28 | 0.28 | 0.02-0.53 | 5.02 | 0.09 | -9.19-19.22 | -0.78 | -0.34 | -1.39 to -0.17 |

***Supplementary Table S8: Regression analysis for key EORTC QLQ-C30 symptom scales at 1 year FU***

|  | **At inclusion** | | | **Male sex** | | | **Age** | | |
| --- | --- | --- | --- | --- | --- | --- | --- | --- | --- |
|  | **b** | **β** | **95% CI** | **b** | **β** | **95% CI** | **b** | **β** | **95% CI** |
| Fatigue | 0.39 | 0.48 | 0.18-0.6 | 1.47 | 0.03 | -11.14-14.08 | 0.73 | 0.35 | 0.18-1.28 |
| Dyspnea | 0.28 | 0.35 | 0.07-0.5 | -5.11 | -0.1 | -18.64-8.44 | 0.47 | 0.21 | -0.12-1.05 |
| Insomnia | 0.13 | 0.15 | -0.11-0.37 | -1.48 | -0.03 | -16.15-13.19 | 0.9 | 0.38 | 0.25-1.56 |

***Supplementary Table S9: Regression analysis for EORTC QLQ-C30 functioning scales at 3 year FU***

|  | **At inclusion** | | | **Male sex** | | | **Age** | | |
| --- | --- | --- | --- | --- | --- | --- | --- | --- | --- |
|  | **b** | **β** | **95% CI** | **b** | **β** | **95% CI** | **b** | **β** | **95% CI** |
| Cognitive functioning | 0.26 | 0.22 | -0.25-0.77 | -2.13 | -0.04 | -24.34-20.07 | -0.84 | -0.41 | -1.75-0.07 |
| Emotional functioning | 0.19 | 0.2 | -0.11-0.49 | 2.27 | 0.05 | -12.94-17.47 | -1.32 | -0.66 | -1.92 to -0.73 |
| Physical functioning | 0.19 | 0.26 | -0.04-0.42 | -0.38 | -0.02 | -6.94-6.17 | -0.54 | -0.66 | -0.8 to -0.28 |
| Role functioning | 0.02 | 0.03 | -0.21-0.26 | 2.64 | 0.08 | -8.29-13.56 | -0.86 | -0.64 | -1.32 to -0.40 |
| Social functioning | -0.12 | -0.1 | -0.50-0.27 | 1.97 | 0.04 | -15.47-19.4 | -1.27 | -0.61 | -2.01 to -0.53 |

***Supplementary Table S10: Regression analysis for key EORTC QLQ-C30 symptom scales at 3 year FU***

|  | **At inclusion** | | | **Male sex** | | | **Age** | | |
| --- | --- | --- | --- | --- | --- | --- | --- | --- | --- |
|  | **b** | **β** | **95% CI** | **b** | **β** | **95% CI** | **b** | **β** | **95% CI** |
| Fatigue | 0.23 | 0.3 | -0.07-0.52 | 1.02 | 0.03 | -14.03-16.07 | 0.74 | 0.48 | 0.12-1.36 |
| Dyspnea | 0.00 | 0.01 | -0.18-0.18 | 2.27 | 0.1 | -9.47-14.01 | 0.14 | 0.14 | -0.32-0.6 |
| Insomnia | 0.19 | 0.23 | -0.06-0.44 | -11.47 | -0.21 | -27.99-5.04 | 1.31 | 0.56 | 0.61-2.01 |

***Supplementary Table S11: Regression analysis for EORTC QLQ-FA12 scales***

|  |  |  | **At inclusion** | | | **Male sex** | | | **Age** | | |
| --- | --- | --- | --- | --- | --- | --- | --- | --- | --- | --- | --- |
|  | **n** | **Year** | **b** | **β** | **95% CI** | **b** | **β** | **95% CI** | **b** | **β** | **95% CI** |
| Physical fatigue | 52 | 1 | 0.34 | 0.38 | 0.12-0.57 | -3.27 | -0.07 | -15.41-8.88 | 0.86 | 0.42 | 0.35-1.37 |
|  | 27 | 3 | 0.33 | 0.33 | -0.03-0.7 | -6.51 | -0.15 | -23.72-10.7 | 0.85 | 0.45 | 0.15-1.54 |
| Emotional fatigue | 52 | 1 | 0.25 | 0.23 | -0.02-0.52 | -2.77 | -0.07 | -13.13-7.59 | 0.76 | 0.43 | 0.31-1.21 |
|  | 27 | 3 | 0.55 | 0.56 | 0.18-0.92 | -0.3 | -0.01 | -15.62-15.02 | 0.46 | 0.25 | -0.17-1.08 |
| Cognitive fatigue | 52 | 1 | 0.34 | 0.31 | 0.03-0.64 | 1.37 | 0.04 | -8.11-10.85 | 0.23 | 0.16 | -0.18-0.64 |
|  | 27 | 3 | 0.46 | 0.36 | -0.09-1.02 | 4.19 | 0.09 | -16.44-24.83 | 0.35 | 0.18 | -0.47-1.16 |

***Supplementary Table S12: EORTC QLQ-C30 global health status and fatigue scale, unadjusted, by treatment arm***

|  |  | **At inclusion** | | | **After N-AVD** | | | **1 year FU** | | | **3 year FU** | | |
| --- | --- | --- | --- | --- | --- | --- | --- | --- | --- | --- | --- | --- | --- |
|  | **Arm** | **n** | **mean** | **95% CI** | **n** | **mean** | **95% CI** | **n** | **mean** | **95% CI** | **N** | **mean** | **95% CI** |
| **GHS** | A | 48 | 60.9 | 54.2-67.6 | 35 | 54.0 | 47.2-60.9 | 28 | 74.4 | 67.7-81.4 | 12 | 82.3 | 74.5-90.1 |
|  | B | 47 | 67.9 | 62.1-73.6 | 27 | 55.6 | 48.0-63.1 | 24 | 78.0 | 70.8-85.1 | 15 | 81.9 | 72.5-91.4 |
| **Fatigue** | A | 49 | 41.0 | 32.2-49.9 | 35 | 59.4 | 50.4-68.3 | 28 | 27.4 | 17.9-36.9 | 12 | 13.0 | 1.8-24.2 |
|  | B | 48 | 37.7 | 29.5-45.9 | 27 | 51.4 | 40.5-62.4 | 24 | 27.1 | 17.4-36.8 | 15 | 14.8 | 3.9-25.7 |

***Supplementary Table S13: Cox regression analyses for time to recovery from fatigue and time to return to work, by treatment arm***

|  | **n** | **Arm B vs A HR (95% CI)** | **Baseline fatigue HR (95% CI)** | **Age HR (95% CI)** | **Male sex HR (95% CI)** |
| --- | --- | --- | --- | --- | --- |
| **TTR-F** | 79 | 0.92 (0.51-1.68) | 0.99 (0.98-1.00) | 0.98 (0.95-1.00) | 1.70 (0.92-3.16) |
| **TTR-W** | 54 | 1.38 (0.73-2.60) | 1.00 (0.99-1.00) | 0.99 (0.95-1.02) | 1.80 (0.97-3.34) |

*Multivariable Cox regression models including treatment arm, baseline fatigue score, age and sex. Arm A (concomitant) and female sex serve as reference categories; hazard ratios for baseline fatigue and age are given per one-point and per one-year increase, respectively. A hazard ratio above 1 indicates a shorter time to the respective event. Abbreviations: CI: confidence interval; HR: hazard ratio; TTR-F: time to recovery from fatigue; TTR-W: time to return to work.*

***Supplementary Table S14: EORTC QLQ-C30 symptom scales and global health status, standardized to reference population and stratified for irHypothyroidism (irHT)***

|  |  | **At inclusion** | | | **After N-AVD** | | | **1 year FU** | | | **3 year FU** | | |
| --- | --- | --- | --- | --- | --- | --- | --- | --- | --- | --- | --- | --- | --- |
|  | **irHT** | **n** | **mean** | **95% CI** | **N** | **mean** | **95% CI** | **N** | **mean** | **95% CI** | **N** | **mean** | **95% CI** |
| Fatigue | Yes | 21 | +24.4 | 9.6-39.2 | 14 | +51.7 | 38.3-65 | 8 | +2.04 | -7.3-11.4 | 4 | -7.68 | -22.8-7.4 |
|  | No | 76 | +21.3 | 14.8-27.9 | 48 | +34.7 | 26.9-42.5 | 44 | +11.3 | 4.7-18.8 | 23 | -2.7 | -11.2-5.8 |
| Nausea and vomiting | Yes | 21 | +5.84 | -2.3-14 | 14 | +30 | 13-47.1 | 8 | -0.02 | -4.9-4.9 | 4 | -2.1 | - |
|  | No | 76 | +0.97 | -1.1-3 | 48 | +20 | 12-28.3 | 44 | +1 | -1.5-3.1 | 23 | -1.4 | -2.9-0.1 |
| Pain | Yes | 21 | +2.7 | -8-13.4 | 14 | +21.4 | 3.8-38.9 | 8 | -8.87 | -28.4-10.7 | 4 | -17.9 | - |
|  | No | 77 | -0.2 | -5.9-5.6 | 49 | +6.6 | -0.7-13.8 | 44 | -8.1 | -14.3 to -1.8 | 23 | -12.5 | -17 to -7.9 |
| Dyspnea | Yes | 21 | +14 | -2.37-30.3 | 13 | +28.03 | 7.63-48.4 | 8 | -4.67 | -17.57-8.2 | 4 | -13 | - |
|  | No | 77 | +9.1 | 1.9-16.3 | 49 | +16.9 | 7.5-26.4 | 44 | +0.3 | -7.7-8.3 | 23 | -7.2 | -12.8 to -1.6 |
| Insomnia | Yes | 21 | +17.93 | 3.54-32.3 | 14 | +32.22 | 12.66-51.8 | 8 | +4.74 | -14.8-24.3 | 4 | -7.07 | -33.6-19.5 |
|  | No | 77 | +11.4 | 4-18.9 | 49 | +23.4 | 13.5-33.2 | 44 | +5.7 | -12.4-13.5 | 23 | 0.5 | -12.4-13.5 |
| Appetite loss | Yes | 21 | +23.7 | 6-41.4 | 14 | +30 | 7.4-52.7 | 8 | -1.9 | -5.2-1.4 | 4 | -3.3 | - |
|  | No | 76 | +15.6 | 9.1-22.1 | 48 | +19.15 | 11.1-28.1 | 44 | +2.8 | -1.6-7.1 | 23 | -0.4 | -4.6-3.8 |
| Constipation | Yes | 21 | +3.9 | -6.7-14.6 | 14 | +15.04 | -1.34-31.4 | 8 | +14.06 | -8.1-36.2 | 4 | +4.33 | -22.2-30.9 |
|  | No | 76 | +1.7 | -2.1-5.5 | 48 | +17.5 | 8.1-27 | 44 | +0.2 | -3.6-4 | 23 | -1.1 | -7.1-4.9 |
| Diarrhea | Yes | 21 | +2.1 | -7.1-11.2 | 14 | +19.5 | -1.07-40.1 | 8 | +6.81 | -6.3-19.9 | 4 | -4.3 | - |
|  | No | 76 | +4.5 | 0.3-8.7 | 47 | +5.6 | 0.3-11 | 44 | +3.7 | -3.2-10.5 | 23 | -1.4 | -5.6-2.8 |
| Financial difficulties | Yes | 21 | +17.2 | 2.7-31.7 | 14 | +24.3 | 8.4-40.3 | 8 | -2.43 | -12.3-7.4 | 4 | -2.4 | -15.7-10.8 |
|  | No | 76 | +14 | 7-21 | 48 | +29.5 | 18.4-40.6 | 44 | +13.1 | 3.2-23 | 23 | +3.5 | -6.6-13.7 |
| Global health status | Yes | 21 | +24 | 12.5-35.5 | 14 | +45.3 | 33.1-57.4 | 8 | +8.5 | -0.79-17.74 | 4 | +8.65 | 2.02-15.3 |
|  | No | 74 | +25.9 | 21.1-30.7 | 48 | +32.3 | 27-37.5 | 44 | +14.7 | 9.3-20.2 | 23 | +7.7 | 0.7-14.6 |

***Supplementary Table S15: EORTC QLQ-C30 functioning scales, standardized to reference population and stratified for irHypothyroidism (irHT)***

|  |  | **At inclusion** | | | **After N-AVD** | | | **1 year FU** | | | **3 year FU** | | |
| --- | --- | --- | --- | --- | --- | --- | --- | --- | --- | --- | --- | --- | --- |
|  | **irHT** | **n** | **mean** | **95% CI** | **N** | **mean** | **95% CI** | **N** | **mean** | **95% CI** | **N** | **mean** | **95% CI** |
| Cognitive functioning | Yes | 21 | -4.76 | -12.15 | 14 | -27.78 | -44.54 to ‑1.02 | 8 | +0.2 | -19.15-19.55 | 4 | +0.2 | -25.19-25.59 |
|  | No | 76 | -1.12 | -5.57-3.34 | 48 | -18.2 | -26.25 to ‑10.15 | 44 | -5.6 | -11.02-0.64 | 23 | -3.24 | -14.58-8.1 |
| Emotional functioning | Yes | 21 | -19 | -31.18 to ‑6.89 | 14 | -20.62 | -32.39 to ‑8.86 | 8 | -0.88 | -12.87-11.11 | 4 | -1.73 | -19.16 to ‑15.7 |
|  | No | 76 | -24.33 | -30.1 to ‑18.59 | 48 | -21.57 | -28.8 to ‑14.34 | 44 | -9.71 | -17 to ‑2.44 | 23 | -5.48 | -16.59-5.62 |
| Physical functioning | Yes | 21 | -1.38 | -8.12-5.36 | 14 | -22.81 | -35.12 to ‑10.5 | 8 | +8.92 | 4.99-12.84 | 4 | +11 | - |
|  | No | 77 | -1.36 | -5.09-2.37 | 49 | -11.31 | -15.71 to ‑6.92 | 44 | +4.33 | 0.84-7.82 | 23 | +6.65 | 2.08-11.2 |
| Role functioning | Yes | 21 | -18.37 | -31.92 to ‑4.81 | 14 | -55.67 | -74.54 to ‑36.79 | 8 | -0.11 | -12.52-12.3 | 4 | +11 | - |
|  | No | 77 | -13.89 | -20.35 to ‑7.44 | 49 | -37.3 | -45.43 to ‑29.17 | 44 | -2.38 | -9.2-4.42 | 23 | +3.03 | -4.46-10.52 |
| Social functioning | Yes | 21 | -4.17 | -15.39 to ‑7.06 | 14 | -21.23 | -38.18 to ‑4.27 | 8 | +19.99 | 10.18-29.8 | 4 | +18.95 | 6.25-31.65 |
|  | No | 76 | 0.2 | -6.1-6.5 | 48 | -16.81 | -25.74 to ‑7.89 | 44 | +8.72 | 0.18-17.26 | 23 | +12.52 | 0.82-24.21 |
